# Research on the Drilling Performance of a Helical Point Micro Drill with Different Geometry Parameters

**DOI:** 10.3390/mi8070208

**Published:** 2017-06-29

**Authors:** Zhiqiang Liang, Suyan Zhang, Xibin Wang, Haixin Guo, Tianfeng Zhou, Li Jiao, Pei Yan

**Affiliations:** Key Laboratory of Fundamental Science for Advanced Machining, Beijing Institute of Technology, 100081 Beijing, China; zsyangela@sina.com (S.Z.); cutting0@bit.edu.cn (X.W.); 18222913930@163.com (H.G.); zhoutf@bit.edu.cn (T.Z.); jiaoli@bit.edu.cn (L.J.); pyan@bit.edu.cn (P.Y.)

**Keywords:** micro-drill, geometry parameters, drilling performance, helical point, stainless steel

## Abstract

During the micro-drilling process of stainless steel, the wear, fracture, and breakage of the micro-drill easily occur. Micro-drill geometry parameters have significant influence on the drilling performance of the micro-drill. Nowadays, the helical point micro-drill is proposed and its improved drilling performance is validated by some researchers. In this study, to analyze the effect of geometry parameters of the helical point micro-drill on drilling performance, the mathematical models of the helical flank and ground flute are proposed, and the cutting lip shape, rake angle, and uncut chip thickness are calculated using MATLAB software. Then, based on the orthogonal tests, nine kinds of micro-drills with different point angles, web thicknesses, and helix angles are fabricated using a six-axis CNC tool grinder, and micro-drilling experiments on 1Cr18Ni9Ti stainless steel are carried out. The drilling force, the burr height, and the hole wall quality are measured and observed. The results show that the point angle is the main contributing factor for the thrust force and burr height, and the web thickness is the main contributing factor for the micro hole wall quality. The increased point angle offers a larger thrust force, but gives rise to a smaller exit burr. A larger web thickness leads to a larger thrust force and burr height, and results in a poor surface quality. With the helix angle increased, the thrust force and burr height decreases, and the surface quality of micro-hole improves. The geometry parameters with a point angle 70°, a point angle of 40°, and web thickness ratio of 0.2 can used to improve the drilling performance of the helical point micro-drill.

## 1. Introduction

Micro-holes have been widely applied in various fields ranging from precision mechanics to advanced electronics [1]. The micro-hole processing method mainly includes the electrical discharge machining [2], laser machining [3], helical milling [4], and drilling. Micro drilling technology is the main method to machine the micro holes precisely and efficiently, because of its widely-machinable materials, high material removal rate, and high machining accuracy. Most of the micro-hole materials are stainless steel, high-strength steel and other difficult-to-cut materials. However, during the micro-drilling process of stainless steel material, serious size effects and difficult chip removal lead to the serious drill wear, fracture, and breakage.

Micro-drill geometry parameters which determine the cutting lip shape, rake angle, uncut chip thickness, and cut width have great influence on the drilling performance, including the drilling force and hole quality [5]. Therefore, many studies have been performed to design and optimize the geometric parameters to improve the drill performance. Fu et al. discussed the influence of the helix angle, web thickness, flute ratio, and primary face angle on the PCB micro-drilling performance, and the results showed that a larger helix angle, web thickness, and flute ratio can improve the drilling performance of a high-aspect-ratio micro-drill [6,7]. Zheng et al. found that the point angle, helix angle, and web thickness had a significant effect on the burr height and roughness of PCB micro-holes, the roughness increased with the increasing point angle and decreased with the increasing helix angle and length of the chisel edge [8,9]. Based on the Taguchi method and response surface methodology, Yoon et al. studied the influence of the helix angle and web thickness on the drilling force and tool wear. The results showed that, for PCB micro-drilling, micro-drill with a helix angle of 42° and a web thickness of 50 μm is the optimal structure [10].

Singh [11] studied the drilling characteristics of UD-GFRP composite laminates, and found that the thrust force increases with the increased point angle, and a 90° point angle causes little hole damage. Utilizing an L12 Taguchi fractional factorial orthogonal array with an analysis of variance (ANOVA), Shyha evaluated the effect of drill geometry on cutting force and tool life when drilling carbon fiber-reinforced plastic (CEPR), and demonstrated that the point angle had a significant effect on the measured outputs, followed by the helix angle [12]. By using a combined simulation and experiment approach, Lauderbaugh analyzed the effect of drill parameters on the exit burr when drilling 2024-T351 aluminum and 7075-T6 aluminum, and reported that the web thickness is consistently a significant factor in burr height [13]. To minimize burr size, a methodology of Taguchi optimization for multi-objective drilling problem was presented by Gaitonde, the result showed that the point angle has a great influence on burr size [14].

Nowadays, helical point micro-drill is proposed by some researchers, and its improved drilling performance is validated compared with planar and conical drill points [15,16]. For the visualization design and optimization of helical-point drill, a numerical modeling and simulation CAD system was presented by Yan based on the helical-point drill model, and the geometric parameters could be modified reasonably according to different drilling requirements [17]. Paul et al. investigated the optimization of the helical-point drill point in order to minimize thrust and torque, and found that the optimized drill had larger rake angles and a smaller point angle [18].

However, the current studies on the design and optimization of micro-drill geometry parameters focus on PCB, aluminum, and other easy-to-cut materials, and the analysis on the stainless steel material is poor. Furthermore, the helical point micro-drill is not widely appreciated and exploited, and its drilling performance with different geometry parameters is scanty. Therefore, this study analyzes the effect of geometry parameters of helical point micro-drill on the drilling performance of stainless steel material. Firstly, the mathematical models of the helical flank and the ground flute of the micro-drill are proposed, and the cutting lip shape, rake angle, and uncut chip thickness of micro-drills with different geometry parameters are calculated by MATLAB software (MathWorks, Natick, MA, USA). Then micro-drills with different point angles, web thicknesses, and helix angles are fabricated by using a six-axis CNC tool grinder, and a serial of micro-drilling experiments on 1Cr18Ni9Ti austenitic stainless steel are carried out. The drilling force, the burr height, and hole wall quality are measured and observed to obtain the optimized micro-drill geometry parameters.

## 2. Mathematical Model of the Helical Point Micro-Drill

### 2.1. Mathematical Model of the Helical Flank of Micro-Drill

**Figure 1:**
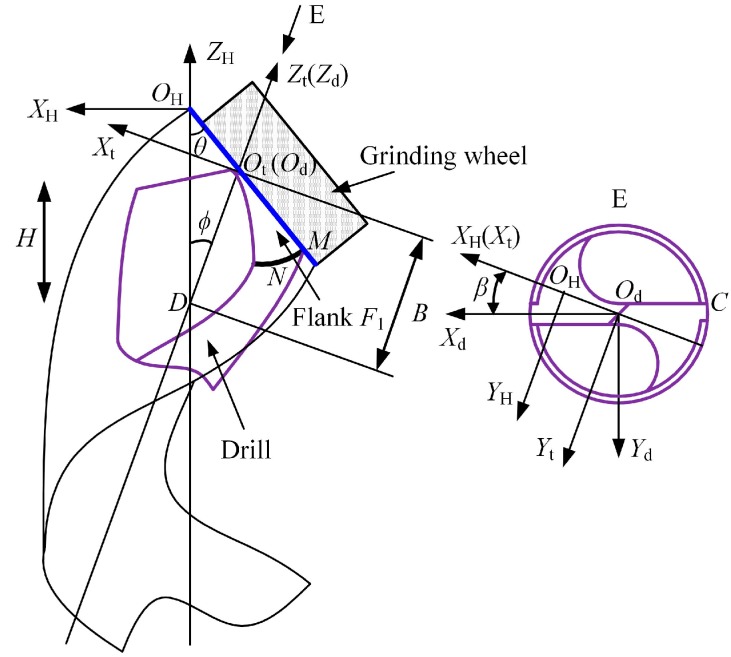
The mathematical model of the flank surface of the helical point micro-drill.

Based on the mathematical model proposed by Zhang [1], the mathematical model of the flank surface of the helical point micro-drill is shown in Figure 1. *X*_d_*Y*_d_*Z*_d_ is the coordinate frame built in the drill, with *O*_d_ located at the drill tip, the *Z*_d_-axis coinciding with the drill axis, and the direction of *X*_d_-axis positioning the *y*-coordinate of the outer corner *C* (*y_c_* = −*t*, 2*t* is web thickness). The equation of the helical flank in system *X*_d_*Y*_d_*Z*_d_ can be expressed as:

(1) $$\begin{array}{l} F_{1}:Z_{d}\cos\phi -B\frac{\sin\phi }{\tan\theta }+\bar{X}\sin\phi +\sqrt{{\left[\bar{X}\cos\phi -\sin\phi \left(Z_{d}+B\right)\right]}^{2}+{\bar{Y}}^{2}}/\tan\theta \\ +\frac{H}{2\pi }\sin^{-1}\left(\bar{Y}/\sqrt{{\left[\bar{X}\cos\phi -\sin\phi \left(Z_{d}+B\right)\right]}^{2}+{\bar{Y}}^{2}}\right)=0 \end{array},$$

where $\bar{X}=X_{d}\cos\beta -Y_{d}\sin\beta$, $\bar{Y}=Y_{d}\cos\beta +X_{d}\sin\beta$, *θ*, *β*, *φ*, *B*, and *H* are grinding parameters of the helical flank. 

Substituting *X*_d_ = −*X*_d_, *Y*_d_ = −*Y*_d_ into the flank equation (Equation (1)), the mathematical model of the flank *F*_2_ (*X*_d_, *Y*_d_, *Z*_d_) = 0 can be obtained and the relationship between the drill geometry parameters $\left(\rho ,\;\psi ,\;\alpha_{fc},\;\alpha_{h,-60^{\circ }}^{R}\right)$ and grinding parameters is (θ, β, ϕ, B, H) [17]:

(2) $$\left\{ \begin{array}{l} \rho =g_{1}(\theta ,\;\beta ,\;\phi ,\;B,\;H)\\ \psi =g_{2}(\theta ,\;\beta ,\;\phi ,\;B,\;H)\\ \alpha_{fc}=g_{3}(\theta ,\;\beta ,\;\phi ,\;B,\;H)\\ \alpha_{h,-60^{\circ }}^{R}=g_{4}(\theta ,\;\beta ,\;\phi ,\;B,\;H) \end{array}\right..$$

### 2.2. Mathematical Model of the Micro-Drill Flute

**Figure 2:**
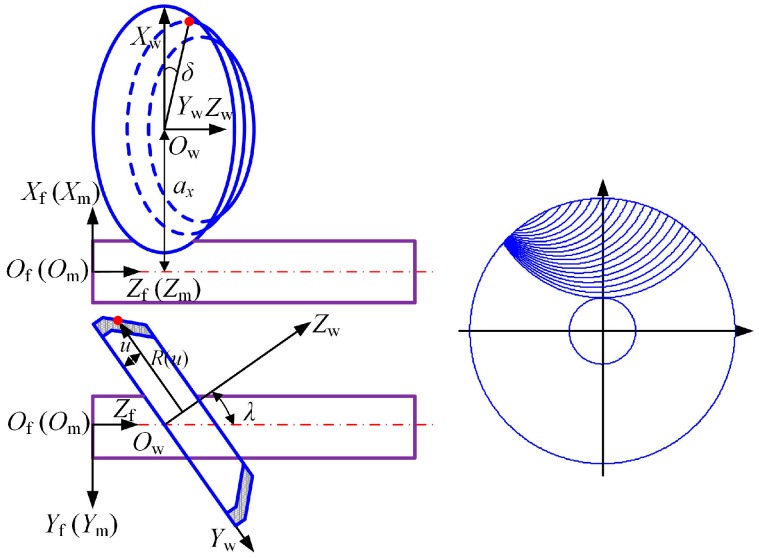
The mathematical model of the micro-drill flute.

The mathematical model of the drill flute is closely bound with the way it is manufactured, and the flute profile largely depends on the grinding parameters, wheel profile, and position. The flute cross-sectional profile can be obtained by the envelope of the cutting paths [19], as shown in Figure 2. The cutting path can be derived as:

(3) $$R_{m}(u,\delta )=\left[\begin{array}{c} X_{\mathrm{Pm}}(u,\delta )\\ Y_{\mathrm{Pm}}(u,\delta ) \end{array}\right]=\left[\begin{array}{c} X_{\mathrm{Pf}}\cos(-Z_{\mathrm{Pf}}\tan(\beta_{0})/r)-Y_{\mathrm{Pf}}\sin(-Z_{\mathrm{Pf}}\tan(\beta_{0})/r)\\ X_{\mathrm{Pf}}\sin(-Z_{\mathrm{Pf}}\tan(\beta_{0})/r)+Y_{\mathrm{Pf}}\cos(-Z_{\mathrm{Pf}}\tan(\beta_{0})/r) \end{array}\right],$$

where, $R_{f}(u,\delta )=\left[\begin{array}{c} X_{\mathrm{Pf}}\\ Y_{\mathrm{Pf}}\\ Z_{\mathrm{Pf}} \end{array}\right]=\left[\begin{array}{c} R(u)\cos\delta +a_{x}\\ R(u)\sin\delta \cos\lambda -u\sin\lambda \\ R(u)\sin\delta \sin\lambda +u\cos\lambda \end{array}\right]$, *u*, and *δ* are parameters of the wheel profile, and *a_x_* and *λ* are parameters of the wheel position.

In order to obtain the mathematical model of the drill flute in system *O*_d_*X*_d_*Y*_d_*Z*_d_, the coordinate points of the flute profile are processed by cubic-spline interpolation, and the equation of the cross-section profile of the drill flute is expressed as: $y_{d}=f_{H}(x_{d})$. The flute surface can be generated by the helical motion of the cross-section profile, so the parameter equation of drill flute is derived as:

(4) $$F_{3}:\{\begin{array}{c} X_{d}=w\cos v-f_{H}(w)\sin v\\ Y_{d}=w\sin v+f_{H}(w)\cos v\\ Z_{d}=rv/\tan\beta_{0} \end{array}.$$

## 3. Geometrical Characteristic of the Helical Point Micro-Drill

To analyze the influence of the micro-drill geometric parameters on the cutting performance, the geometrical characteristic of micro-drill with different parameters should be obtained and discussed firstly. The drill geometric parameters have a significant influence on the cutting lip shape, the rake angle, the uncut chip thickness, and the cut width, and finally affect the chip deformation and drilling force. Therefore, in this paper, the dynamic rake angle distribution along the chisel edge and cutting lip are calculated, and the cutting lip shape and the uncut chip thickness are also derived.

For any point *Q* on the cutting lip, the position vector is **q** = (*x*, *y*, *z*), and the coordinate (*x*, *y*, *z*) is obtained by two simultaneous equations *F*_1_ (*X*_d_, *Y*_d_, *Z*_d_) = 0 and *F*_3_ (*X*_d_, *Y*_d_, *Z*_d_) = 0. The resultant cutting velocity *V*_e_ of point *Q* is expressed as: *V*_e_ = (−2π*ny*/60, 2π*nx*/60, *nf*/60), *n* is the rotation speed (r/min), and *f* is the feed rate (mm/r).

The vectors **g**, **h** are the unit vectors normal to the rake and clearance surface, **g** = (*g_x_*, *g_y_*, *g_z_*) = (∂*F*_1_/∂*x*, ∂*F*_1_/∂*y*, ∂*F*_1_/∂*z*), **h** = (*h_x_*, *h_y_*, *h_z_*) = (∂*F*_3_/∂*x*, ∂*F*_3_/∂*y*, ∂*F*_3_/∂*z*), so the unit vector **b** along the cutting lip can be expressed as: **b** = (**g** × **h**)/|**g** × **h**|, and the unit vectors normal to the working reference plane, cutting edge plane, and orthogonal plane is:

(5) $$r=\left(r_{x},r_{y},r_{z}\text{​}\right)=\frac{V_{e}}{\left|V_{e}\right|},\;s=\left(s_{x},s_{y},s_{z}\text{​}\right)=\frac{V_{e}\times b}{\left|V_{e}\times b\right|},\;o=\left(o_{x},o_{y},o_{z}\text{​}\right)=s\times r.$$

Therefore, the dynamic rake angle *γ*_oe_ and tool cutting edge angle *κ*_re_ can be obtanined [20]:

(6) $$\gamma_{oe}=\tan^{-1}\left(\left|\begin{array}{ccc} g_{x} & g_{y} & g_{z}\\ r_{x} & r_{y} & r_{z}\\ o_{x} & o_{y} & o_{z} \end{array}\right|/\left(g\times o)\cdot (r\times o\right)\right),\;\kappa_{re}=\cos^{-1}\left(o\cdot \frac{k-(k\cdot r)r}{\left|k-(k\cdot r)r\right|}\right),\;k=\left(0,\;0,\;1\right).$$

The calculation equation of the uncut chip thickness is expressed as:

(7) $$a_{c}=\frac{1}{2}f\cos(\sin^{-1}(k\cdot r))\cdot \sin\kappa_{\mathrm{re}}.$$

Based on the mathematical model and geometric principle, a program in MATLAB is used to calculate the numerical solution. The geometric parameters of micro-drills are listed in Table 1. The geometric and position parameters of the wheel for grinding the flute are listed in Table 2.

**Figure 3:**
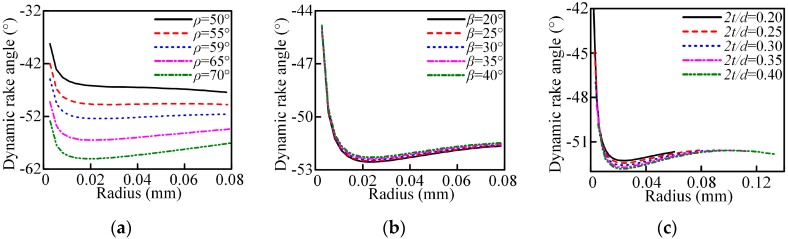
Dynamic rake angle distribution along the chisel edge with *n* = 14,000 r/min, *f* = 0.003 mm/r: (**a**) point angle; (**b**) helix angle; (**c**) web thickness ratio.

**Figure 4:**
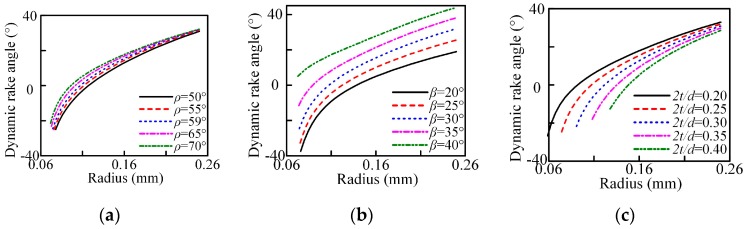
Dynamic rake angle distribution along the cutting lip with *n* = 14,000 r/min, *f* = 0.003 mm/r: (**a**) point angle; (**b**) helix angle; and (**c**) web thickness ratio.

**Figure 5:**
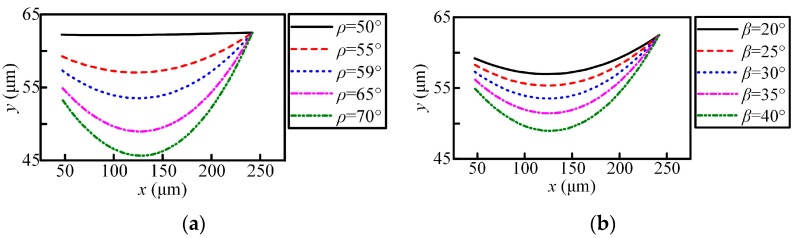
The shape of the cutting lip: (**a**) point angle; and (**b**) helix angle.

**Figure 6:**
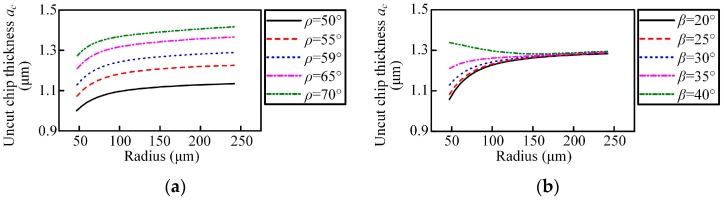
The uncut chip thickness along the cutting lip with *n* = 14,000 r/min, *f* = 0.003 mm/r: (**a**) point angle; and (**b**) helix angle.

The dynamic rake angle distribution along the chisel edge and cutting lip are shown in Figure 3 and Figure 4. The cutting lip shape and the corresponding uncut chip thickness are shown in Figure 5 and Figure 6. With the point angle increased, the dynamic rake angle along the cutting lip increases by a small margin (shown in Figure 4a), but the dynamic rake angle distribution along the chisel edge decreases evidently (shown in Figure 3a), which will enlarge the chip deformation and increase the thrust force. Furthermore, Figure 5a shows that the curvature of the cutting lip shape becomes larger with the increased point angle, which leads to the increase of the uncut chip thickness (shown in Figure 6a). However, the cut width becomes smaller when the point angle becomes larger.

The helix angle has a significant impact on the rake angle along the cutting lip, the higher helix angle leads to a larger rake angle, as shown in Figure 4b. Figure 5b shows that the curvature of the cutting lip shape becomes larger with the increased helix angle, but the uncut chip thickness has no obvious change (as shown in Figure 6b).

Furthermore, an increased web thickness ratio leads to the increase of the non-efficient cutting edge length and reduces the dynamic rake angle of the cutting lip, which will enlarge the chip deformation and increase the thrust force.

## 4. Grinding Experiment of Helical Point Micro-Drills with Different Geometry Parameters

**Figure 7:**
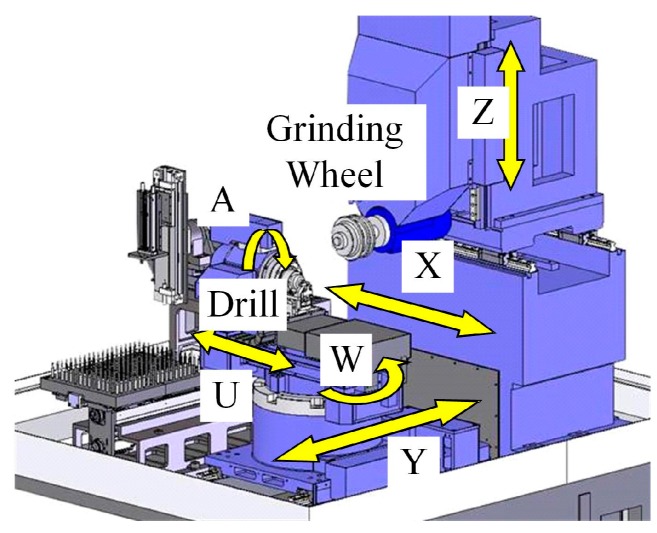
The six-axis CNC grinding machine.

**Figure 8:**
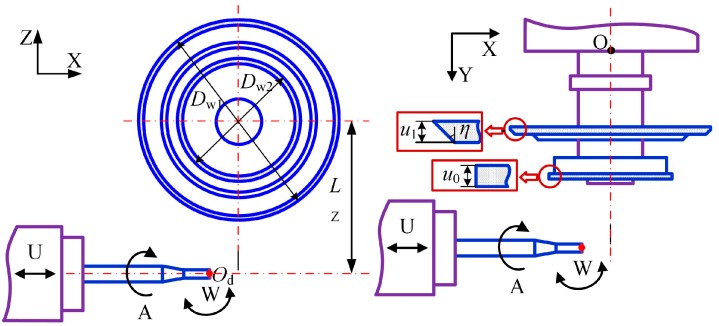
The geometry and configuration of the grinding wheels.

Based on the grinding process of the helical flank and drill flute presented by Zhang [1,19], the micro-drill is fabricated using a six-axis CNC grinding machine (CNS7d by Makino Seiki Co., Ltd., Kanagawa, Japan), as shown in Figure 7. The geometry of the grinding wheels and the configuration of the two wheels are shown in Figure 8. The helical flank is ground by Wheel 1, with *D*_w1_ = 80 mm, *u*_0_ = 3 mm. The drill flute is ground by Wheel 2, with *D*_w2_ =135 mm, *u*_1_ = 4 mm, *η* = 45°.

**Figure 9:**
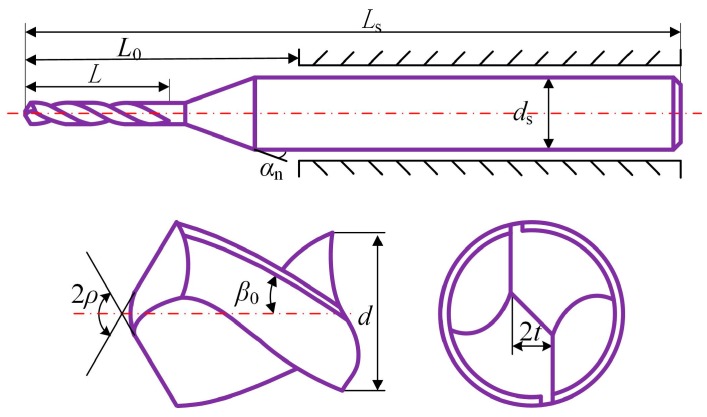
The structure of the micro-drill.

**Figure 10:**
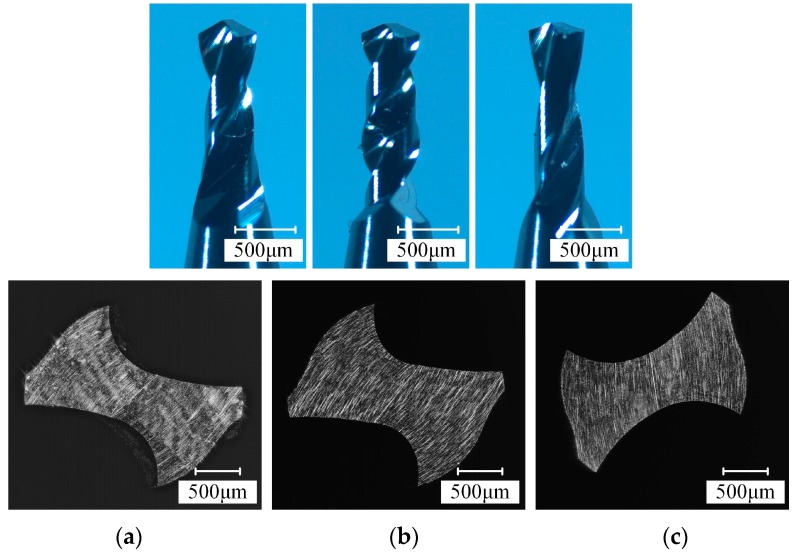
The fabricated result of helical point micro-drills. (**a**) *ρ* = 59°, *β* = 30°, 2*t*/*d* = 0.25; and (**b**) *ρ* = 65°, *β* = 40°, 2*t*/*d* = 0.3; (**c**) *ρ* = 70°, *β* = 20°, 2*t*/*d* = 0.25.

To analyze the influence of drill geometry parameters on the micro-drilling performance, the orthogonal tests are designed according to the factor level table listed in Table 3. Then nine kinds of micro-drills with different point angles, web thickness ratios, and helix angles of the flute are fabricated. The structure of the micro-drill is shown in Figure 9, and the corresponding geometric parameters are listed in Table 4. The fabricated result of the micro-drills are shown in Figure 10.

## 5. Drilling Experiment of Helical Point Micro-Drill with Different Geometry Parameters

**Figure 11:**
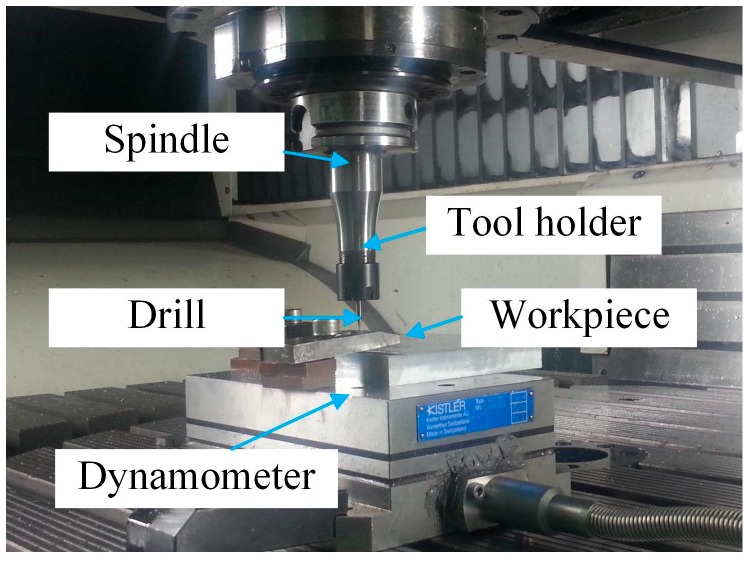
The drilling experiment setup on a DMG machining center.

A series of drilling experiments with different micro-drills are carried out on DMG machining center (DMU80 monoBLOCK by DMG MORI Co., Ltd., Germany), shown in Figure 11. The workpiece material is 1Cr18Ni9Ti austenitic stainless steel and the experiment parameters are set to the spindle speed 14,000 r/min, with a feed rate of 0.003 mm/r.

**Figure 12:**
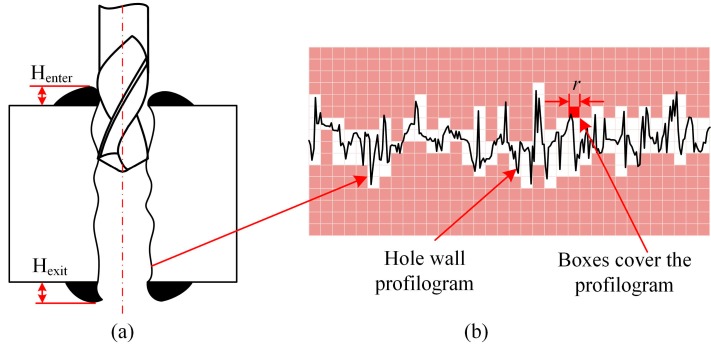
Schematic diagram of burr height measurement and the 2D box-counting method. (**a**) The burr height measurement (**b**) the 2D box-counting method.

The drilling force is measured using a Kistler piezoelectric dynamometer model 9257B. The burr height and hole wall quality are measured and observed using 3D laser scanning microscope (VK-100 by Keyence Co., Ltd., Osaka, Japan) and scanning electron microscope (S4800 by HITACHI Co., Ltd., Tokyo, Japan), the measurement method of burr height *H*_enter_ and *H*_exit_ is shown in Figure 12a.

Moreover, conventional evaluation methods of surface topography, such as statistical parameter Ra, strongly depend on actually sampling and the scan lengths, and cannot evalute the surface quality precisely in the meso-scale [21]. However, many machined surfaces, such as those processed by turning, drilling, and grinding, which have the property of self similarity or self-affinity, can be characterised with fractal geometry [22]. The fractal dimension is more effective to evaluate the surface quality than the conventional method at the meso-scale [23]. Therefore, in this paper, the hole wall quality are evaluated with fractal dimension *D_L_* generated by the 2D box counting method shown in Figure 12b and discussed in the literature [21]. The equation of fractal dimension *D_L_* is:

(8) $$D_{L}=-\lim\frac{\ln N(r)}{\ln r},$$

where, *N*(*r*) is the minimal number of boxes covering the fractal object, and *r* is the size of the box.

## 6. Drilling Result of a Helical Point Micro-Drill with Different Geometry Parameters

The experiment results, including thrust force, burr height, and hole wall qualty, with different drill geometries are listed in Table 5. Range analysis is used to evalute the main or minor contributory factor, and the results are listed in Table 6.

### 6.1. Drilling Force of Micro-Drills with Different Geometry Parameters

**Figure 13:**
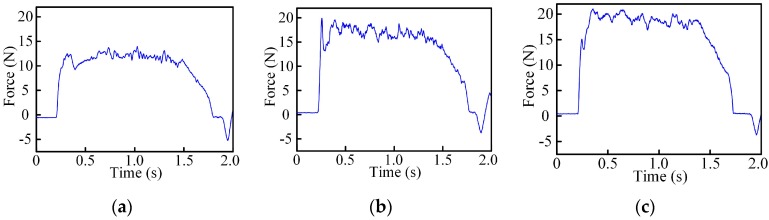
Thrust force of micro-drills with different geometric parameters. (**a**) *ρ* = 59°, *β* = 40°, 2*t*/*d* = 0.25; (**b**) *ρ* = 65°, *β* = 20°, 2*t*/*d* = 0.25; (**c**) *ρ* = 70°, *β* = 30°, 2*t*/*d* = 0.3.

**Figure 14:**
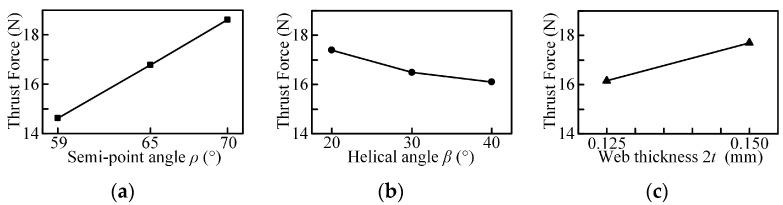
Main effect plot for mean of thrust force: (**a**) point angle; (**b**) helical angle; (**c**) web thickness.

The thrust force for micro-drills with different geometric parameters are shown in Figure 13, which clearly reveals the penetration phases. Analysis of variance (ANOVA) by Minitab are empolyed to assess the relative merits of factors and the sensitivity of the various level. The main effect plots and associated ANOVA results for thrust force are shown in Figure 14 and Table 7.

The results show that the thrust force increases with the increase of the point angle and web thickness, and decreases with the increase of helix angle. As the rake angle is a significant factor for the material plastic deformation, it has a great effect on the thrust force. The increased point angle offers a smaller dynamic rake angle distribution along the chisel edge (see Figure 3a), which will enlarge the chip deformation and increase the thrust force. Furthermore, as the point angle increases, the proportion of the axial force to the cutting force increases, so the total thrust force increases. With the helix angle increased, the cutting width increases which will enlarge the cutting force due to the curvature of the cutting lip shape becomes larger (see Figure 5b). However, the dynamic rake angle along the cutting lip increases (see Figure 4b), which will lead to the thrust force decrease. In general, a larger helix angle give rise to a smaller thrust force. Additionally, the contribution of the chisel edge to the thrust force is tremendous, an increased web thickness leads to the increase of the non-efficient cutting edge length and reduces the dynamic rake angle of cutting lip, as shown in Figure 4c, resulting in a higher thrust force.

Furthermore, the statistical analysis shows that the point angle has a significant influence on thrust force in the selected range, since the value of F is larger than the critical value shown in Table 7, and the point angle is the main contributory factor followed by the web thickness, while the helix angle has a moderate effect on the thrust force based on the range analysis results shown in Table 6.

### 6.2. Burr Height of Micro-Drills with Different Geometry Parameters

**Figure 15:**
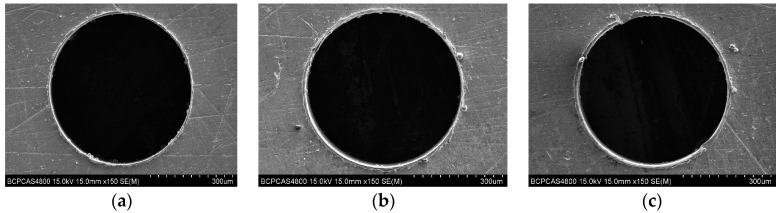
Entrance burr for helical point micro-drills with different geometric parameters. (**a**) *ρ* = 59°, *β* = 20°, 2*t*/*d* = 0.3; and (**b**) *ρ* = 65°, *β* = 30°, 2*t*/*d* = 0.25; (**c**) *ρ* = 70°, *β* = 40°, 2*t*/*d* = 0.25.

**Figure 16:**
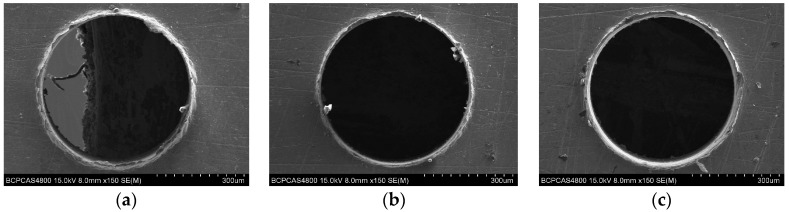
Exit burr for helical point micro-drills with different geometric parameters. (**a**) *ρ* = 59°, *β* = 20°, 2*t*/*d* = 0.3; and (**b**) *ρ* = 65°, *β* = 20°, 2*t*/*d* = 0.25; (**c**) *ρ* = 70°, *β* = 30°, 2*t*/*d* = 0.3.

Burrs are formed during the drilling process on the entrance and exit surface of the micro-holes, as a result of plastic deformation. Burrs will result in the deterioration of micro-hole quality, and reduce the product durability and precision. The entrance burr and exit burr of micro-holes are shown in Figure 15 and Figure 16. The entrance burr type is the same, basically, as a result of a tearing, bending action followed by shearing or lateral extrusion. For the exit burr, the Poisson burr forms (Figure 16a) due to the material bulging to the sides when it is compressed, until permanent plastic deformation occurs and the rollover burr forms (Figure 16c) as a result of the bending action rather than shearing of the chip at the end of drilling.

**Figure 17:**
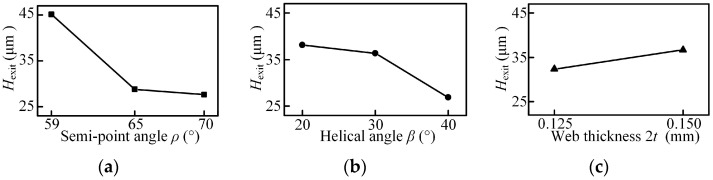
Main effects plot for the mean of the exit burr height: (**a**) point angle; (**b**) helical angle; (**c**) web thickness.

**Figure 18:**
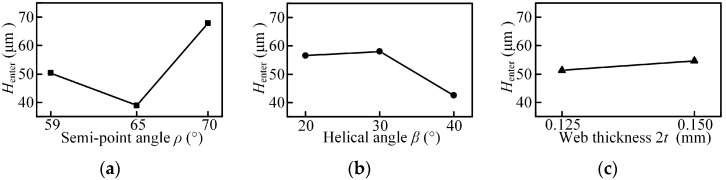
Main effects plot for the mean of the entrance burr height: (**a**) point angle; (**b**) helical angle; (**c**) web thickness.

The main effects plots and associated ANOVA results for the burr height are shown in Figure 17 and Figure 18 and Table 8 and Table 9. The exit burr height decreases with the increased point angle and helix angle, and increases with the increase in web thickness. When the micro drill exits the workpiece, the higher point angle keeps the work material under tensile stress, allowing the material to cut very easily, instead of pushing it out of the workpiece, changing the chip flow direction. This results in the strain at the drill point being smaller than that in the out corner and chip moves at an earlier possible time to avoid work hardening, thus giving rise to a smaller burr. For the helix angle and web thickness, the variation changes the dynamic rake angle distribution (Figure 4b,c) and the chip deformation, then induces the variation of the burr height. The ANOVA results in Table 8 show that the three parameters all have great influence on the exit burr height in the selected range due to the larger value of F. Furthermore, from range analysis results, shown in Table 6, it can be seen that the point angle has the most significant effect followed by the helix angle, and web thickness is also crucial for the exit burr height. However, the entrance burr height is not linear with the point angle and helix angle in the selected range, and the point angle and helix angle have significant influence on it in terms of the value of *F*, and the main contributing factor is also the point angle.

### 6.3. Machining Quality of Mirco Hole by Micro-Drills with Different Geometry Parameters

**Figure 19:**
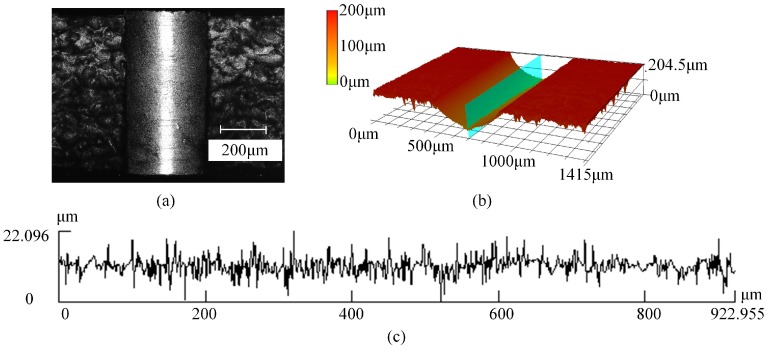
The hole wall morphology. (**a**) The hole wall morphology; (**b**) the 3D laser scanning morphology; (**c**) the hole wall profile.

The quality of micro-holes wall determines the performance of micro-holes, and it is a significant factor to evaluate the drilling quality. The hole wall morphology is shown in Figure 19a, and one profile is obtained by the 3D laser scanning microscope show in Figure 19c.

**Figure 20:**
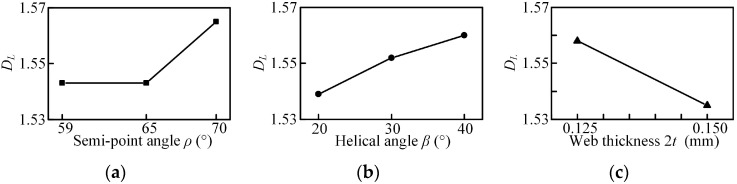
Main effects plot for the mean of the fractal dimension: (**a**) point angle; (**b**) helical angle; (**c**) web thickness.

Based on the profile obtained by laser scanning microscope, fractal dimension *D_L_* are generated by the 2D box counting method using MATLAB. The experimental results and its range analysis are listed in Table 5 and Table 6. The main effects plots and associated ANOVA results for *D_L_* are shown in Figure 20 and Table 10. When the helix angle and web thickness increases, the fractal dimension increases and decreases, respectively. However, it is not linear with the point angle in the selected range. The statistical analysis shows that the web thickness has significant influence on fractal dimension *D_L_* in the selected range since the value of F is larger than the critical value, and it is the main contributory factor followed by the point angle and helix angle shown in Table 6.

When the helix angle increases, the chip evacuation capacity improves and the friction between the chip and the hole wall weakens. Thus, a larger helix angle gives rise to a higher surface quality. With the web thickness increased, the chip space reduces and the chip evacuation capacity reduces. Then a larger web thickness leads to a poor surface quality. Moreover, it should be noted that the thrust force increases with the increase of web thickness, decreases with the increase of helix angle shown in Figure 14, and the poor hole quality may be due to the larger drilling force.

## 7. Conclusions

This study analyzes the effect of the geometry parameters of a helix micro-drill on the drilling performance of stainless steel material. The mathematical models of the helix flank, and the ground flute of the micro-drill are proposed, and the cutting lip shape, rake angle, and uncut chip thickness of micro-drills with different geometry parameters are calculated. Using nine micro-drills with different point angles, web thicknesses, and helix angles, a serial of micro-drilling experiments on 1Cr18Ni9Ti austenitic stainless steel was carried out. By discussing the drilling force, the burr height, and hole wall quality, some conclusions can be summarized as follows:

(1) Within a certain range of geometry parameters, the point angle is the main contributing factor for the thrust force followed by web thickness, while the helix angle has a moderate effect. For the burr height, the point angle have the most significant effect followed by helix angle, and web thickness are also crucial for the exit burr height. For the hole quality, the web thickness is the main contributory factor followed by the point angle and helix angle.
(2) The increased point angle offers a smaller dynamic rake angle distribution along the chisel edge and a larger proportion of the axial force to the total cutting force, which will enlarge the thrust force. However, the higher point angle gives rise to a smaller exit burr.
(3) The increased web thickness leads to the increase of the non-efficient cutting edge length and reduces the dynamic rake angle of cutting lip, resulting in a higher thrust force and burr height, and brings down the chip evacuation capacity, resulting in a poor surface quality.
(4) With the helix angle increased, the dynamic rake angle along the cutting lip increases which will lead to the thrust force and burr height decrease, and the chip evacuation capacity improves which will weaken the friction between the chip and the hole wall and give rise to a higher surface quality.
(5) The geometry parameters with point angle 70°, point angle 40°, and web thickness ratio 0.2 can be used to improve the drilling performance of a helical point micro-drill.

## Figures and Tables

**Table 1:** The geometric parameters of the micro-drill.

Parameters	Value
Drill Diameters *d* (mm)	0.5
Web Thickness Ratio	0.2, 0.25, 0.3, 0.35, 0.4
Helix Angle of the Flute *β*_0_ (°)	20, 25, 30, 35, 40
Semi-Point Angle *ρ* (°)	50, 55, 59, 65, 70
Chisel Edge Angle *ψ* (°)	55
Lip Clearance Angle *α_fc_* (°)	12
Heel Clearance Angle $\alpha_{h,-60^{{}^{\circ}}}^{R}$ (°)	15

**Table 2:** The wheel profile and position parameters.

Parameters	Value
Installation Angle *λ* (°)	60
Distance *a_x_* (mm)	67.5625
Wheel Radius *R*_0_ (mm)	67.5
Wheel Thickness *u*_1_ (mm)	4
Wheel Inclined Angle *η* (°)	45

**Table 3:** The factor level table of micro drill geometry parameters.

Level	Semi-Point Angle *ρ* (°)	Web Thickness Ratio 2*t*/*d*	Helix Angle *β*_0_ (°)
1	59	0.25*d*	20
2	65	0.3*d*	30
3	70	-	40

**Table 4:** Geometric parameters of helical point micro-drills.

No.	1	2	3	4	5	6	7	8	9
Semi-point angle *ρ* (°)	59	59	59	65	65	65	70	70	70
Helix angle *β*_0_ (°)	20	30	40	20	30	40	20	30	40
Web thickness 2*t* (mm)	0.15	0.125	0.125	0.125	0.125	0.15	0.125	0.15	0.125
Drill diameter *d* (mm)	0.5	Drill shank diameter *d*_s_ (mm)			3	Drill neck angle *α*_n_ (°)			10
Flute length *L* (mm)	1.5	Drill whole length *L*_s_ (mm)			50	Length of the drill overhang out of the tool holder *L*_0_ (mm)			12

**Table 5:** Thrust force, burr heights, and hole wall quality with different drill geometry parameters.

No.	*ρ* (°)	*β* (°)	2*t* (mm)	Thrust Force (N)	*H*_enter_ (μm)	*H*_exit_ (μm)	Fractal Dimension *D_L_*
1	59	20	0.150	16.44	58.10	52.02	1.51179
2	59	30	0.125	14.53	56.87	46.48	1.55892
3	59	40	0.125	12.88	35.99	36.95	1.55841
4	65	20	0.125	16.94	43.57	33.30	1.54190
5	65	30	0.125	15.85	42.38	28.93	1.54540
6	65	40	0.150	17.53	30.79	24.13	1.54188
7	70	20	0.125	18.83	68.13	28.91	1.56302
8	70	30	0.150	19.12	74.96	33.68	1.55019
9	70	40	0.125	17.88	60.67	20.32	1.58085

**Table 6:** The range analysis results with different drill geometry parameters.

Geometry Parameters	Level	Thrust Force (N)	*H*_enter_ (μm)	*H*_exit_ (μm)	Fractal Dimension *D_L_*
Semi-point angle *ρ*	Level 1	14.62	50.32	45.15	1.543039
	Level 2	16.77	38.91	28.79	1.54306
	Level 3	18.61	67.92	27.64	1.564687
	Range	3.99	29.01	17.51	0.021647
Helix angle *β*	Level 1	17.40	56.60	38.08	1.538903
	Level 2	16.50	58.07	36.36	1.551503
	Level 3	16.10	42.48	27.13	1.56038
	Range	1.31	15.59	10.94	0.021477
Web thickness 2*t*	Level 1	16.15	51.27	32.48	1.558083
	Level 2	17.70	54.62	36.61	1.534619
	Range	1.55	3.35	4.13	0.023464

**Table 7:** ANOVA results for thrust force.

Source of Variance	DF	Seq SS	Adj SS	Adj MS	F	F-Test *
Semi-point angle	2	23.9502	23.9502	11.9751	30.4	9.55
Helix angle	2	2.6866	2.6866	1.3433	1.3433	9.55
Web thickness	1	4.7749	4.7749	4.7749	12.15	10.1
Error	3	1.1788	1.1788	0.3929	-	-
Total	8	32.5906	-	-	-	-

DF: Degrees of freedom SS: Sum of squares MS: Mean of squares. * Value of F variable at 95% confidence level.

**Table 8:** ANOVA results for the exit burr height.

Source of Variance	DF	Seq SS	Adj SS	Adj MS	F	F-Test *
Semi-point angle	2	575.76	575.76	287.88	108.07	9.55
Helix angle	2	207.87	207.87	103.93	39.02	9.55
Web thickness	1	34.05	34.05	34.05	12.78	10.1
Error	3	7.99	7.99	2.66	-	-
Total	8	825.66	-	-	-	-

DF: Degrees of freedom SS: Sum of squares MS: Mean of squares. * Value of F variable at a 95% confidence level.

**Table 9:** ANOVA results for the entrance burr height.

Source of Variance	DF	Seq SS	Adj SS	Adj MS	F	F-Test *
Semi-point angle	2	1281.11	1281.11	640.56	43.94	9.55
Helix angle	2	444.28	444.28	222.14	15.24	9.55
Web thickness	1	22.41	22.41	22.41	1.54	10.1
Error	3	43.74	43.74	14.58	-	-
Total	8	1791.55	-	-	-	-

DF: Degrees of freedom SS: Sum of squares MS: Mean of squares. * Value of F variable at a 95% confidence level.

**Table 10:** ANOVA results for the fractal dimension.

Source of Variance	DF	Seq SS	Adj SS	Adj MS	F	F-Test *
Semi-point angle	2	0.0009363	0.0009363	0.0004682	9.52	9.55
Helix angle	2	0.0006989	0.0006989	0.0003494	7.11	9.55
Web thickness	1	0.0011011	0.0011011	0.0011011	22.39	10.1
Error	3	0.0001475	0.0001475	0.0000492	-	-
Total	8	0.0028838	-	-	-	-

DF: Degrees of freedom SS: Sum of squares MS: Mean of squares. * Value of F variable at 95% confidence level.
